# Genotypic variation in an ecologically important parasite is associated with host species, lake and spore size

**DOI:** 10.1017/S0031182021000949

**Published:** 2021-09

**Authors:** Clara L. Shaw, Rebecca Bilich, Bruce O'Brien, Carla E. Cáceres, Spencer R. Hall, Timothy Y. James, Meghan A. Duffy

**Affiliations:** 1 Department of Ecology & Evolutionary Biology, University of Michigan, Ann Arbor, MI 48109, USA; 2 Department of Evolution, Ecology, & Behavior, School of Integrative Biology, University of Illinois Urbana-Champaign, Urbana, IL 61801, USA; 3 Department of Biology, Indiana University, Bloomington, IN47405, USA

**Keywords:** *Ceriodaphnia*, *Daphnia*, fungal diversity, *Metschnikowia bicuspidata*, multihost, parasite, pathogen, spillover

## Abstract

Genetic variation in parasites has important consequences for host–parasite interactions. Prior studies of the ecologically important parasite *Metschnikowia bicuspidata* have suggested low genetic variation in the species. Here, we collected *M. bicuspidata* from two host species (*Daphnia dentifera* and *Ceriodaphnia dubia*) and two regions (Michigan and Indiana, USA). Within a lake, outbreaks tended to occur in one host species but not the other. Using microsatellite markers, we identified six parasite genotypes grouped within three distinct clades, one of which was rare. Of the two main clades, one was generally associated with *D. dentifera,* with lakes in both regions containing a single genotype. The other *M. bicuspidata* clade was mainly associated with *C. dubia*, with a different genotype dominating in each region. Despite these associations, both *D. dentifera-* and *C. dubia*-associated genotypes were found infecting both hosts in lakes. However, in lab experiments, the *D. dentifera*-associated genotype infected both *D. dentifera* and *C. dubia*, but the *C. dubia*-associated genotype, which had spores that were approximately 30% smaller, did not infect *D. dentifera.* We hypothesize that variation in spore size might help explain patterns of cross-species transmission. Future studies exploring the causes and consequences of variation in spore size may help explain patterns of infection and the maintenance of genotypic diversity in this ecologically important system.

## Graphical abstract

**Figure:**
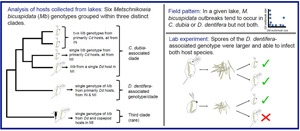

## Introduction

Most parasite species contain substantial diversity (Thompson and Lymbery, 1990), and one of the grand challenges in understanding the evolution of infectious diseases is to understand what promotes this genotype diversity (Metcalf *et al*., 2015). Genetic variation within parasites could lead to variation in infectivity (e.g. Luijckx *et al*., 2011; Thrall *et al*., 2012; Koskella, 2014), virulence (e.g. Morrison *et al*., 2010; Hawley *et al*., 2013; Audebert *et al*., 2020), and other important traits, such as the ability to survive and disperse in the environment (e.g. Tack *et al*., 2014; Mahmud *et al*., 2017; Rogalski and Duffy, 2020). Thus, not only is genetic variation within parasites common, it is also important to the ecology and evolution of host-parasite systems.

Although genetic variation is common and critical for predicting parasite evolution, it is not universal. Even just considering fungal parasites, some are highly diverse (e.g. the biocontrol agent *Beauveria* (Serna-Domínguez *et al*., 2019)), whereas others have extremely low diversity (e.g. *Batrachochytrium dendrobatidis* (James *et al*., 2009), *Geomyces destructans* (Ren *et al*., 2012), *Raffaelea lauricola* (Wuest *et al*., 2017)). Low genetic diversity equates to low effective population size, and may result from recent, rapid geographic spread or clonal reproduction (e.g. Leopardi *et al*., 2015; O'Hanlon *et al*., 2018). However, in other cases, diversity of a parasite is surprisingly low even in systems where the parasite is not thought to have recently invaded new hosts and habitats. One example of this is the ecologically important host-parasite system comprised of the fungus *Metschnikowia bicuspidata* and its zooplankton (daphniid) hosts, where infections can reach high prevalences (~60% of the population with late stage infections at the peak of large outbreaks (Shaw *et al*., 2020)). Intriguingly, research from the 1880s in Europe (Metschnikoff, 1884) and 1970s in the USA (Green, 1974) suggests that *M. bicuspidata* has likely had a world-wide distribution for centuries.

Given its widespread geographic distribution and high prevalence within populations, it is surprising that prior studies have failed to find significant intraspecific variation in *M. bicuspidata*. Parasites collected from different lakes and in different years did not differ in their infectivity or virulence (Duffy and Sivars-Becker, 2007; Searle *et al*., 2015); parasite populations did not respond to artificial selection on (a) infectivity or virulence (Duffy and Sivars-Becker, 2007), (b) within host growth rate (Auld *et al*., 2014), or (c) fungicide resistance (Cuco *et al*., 2020); and comparisons of the SSU, ITS, and partial LSU regions found identical sequences for *M. bicuspidata* collected on different continents from different host species (Wolinska *et al*., 2009). However, these studies were not designed to characterize diversity across regions and hosts. First, the studies on phenotypes (Duffy and Sivars-Becker, 2007; Auld *et al*., 2014; Searle *et al*., 2015; Cuco *et al*., 2020) used *M. bicuspidata* collected from a single region in a single host, but evolutionary forces could generate variation between regions and hosts. Second, the genetic study (Wolinska *et al*., 2009) used relatively conserved loci, which often cannot separate geographic populations or even species for certain fungal taxa. Thus, broader sampling with more sensitive markers might uncover diversity.

We sought to uncover diversity in *M. bicuspidata* by genotyping parasites at microsatellite loci, which are generally more variable than the previously assayed SSU, ITS, and LSU loci (Chistiakov *et al*., 2006) and by collecting samples from two regions and from two host species. In particular, we hypothesized that *M. bicuspidata* genotypes might differ across host species because, while *M. bicuspidata* can infect multiple hosts (Auld *et al*., 2017), when two host species co-occur, it is common to see an outbreak in one host species but not the other. This is especially true in our studies of populations dominated by *Daphnia dentifera* and *Ceriodaphnia dubia* (data presented below). In prior studies, we have found that *C. dubia* is largely resistant to infections with *M. bicuspidata* isolated from *D. dentifera* (Strauss *et al*., 2015; Auld *et al*., 2017). These hosts vary substantially in adult body size (Dodson *et al*., 2010), and we had observed that *M. bicuspidata* spores in smaller-bodied hosts such as *C. dubia* were often notably smaller than those seen in *D. dentifera* in natural infections. Together, this led us to hypothesize that different host species harbour previously unseen variation in *M. bicuspidata*, and that this among-host variation might be associated with key parasite traits.

We conducted a study aimed at quantifying genetic variation in this ecologically important parasite. First, we monitored *M. bicuspidata* prevalence in two host species, *C. dubia* and *D. dentifera.* Second, we developed microsatellite markers and, with these, quantified intraspecific variation in *M. bicuspidata* by genotyping parasites in two infected hosts species (*C. dubia* and *D. dentifera*) collected from multiple lakes in two regions (Michigan (MI) and Indiana (IN), USA). Third, we carried out a lab experiment in which we (1) assessed the ability of parasites isolated from one host species to infect the other host species, (2) quantified spore yield within infected hosts, and (3) measured spore size, a trait that we hypothesized might be associated with the ability to infect different hosts. Overall, we found that outbreaks tend to occur in one host species or the other but not both simultaneously, that there is significant genetic variation in *M. bicuspidata*, and that this variation is associated with the ability to infect different host species, spore yield within infected hosts, and spore size.

## Materials and methods

### Study system

Zooplankton communities experience outbreaks of *M. bicuspidata* in late summer and autumn (Shaw *et al*., 2020). Grazing hosts consume infective, needle-shaped spores floating in the water; infection occurs if these spores pierce through the gut epithelium and are not successfully thwarted by the host immune response (Metschnikoff, 1884; Stewart Merrill and Cáceres, 2018; Stewart Merrill *et al*., 2020). The parasite replicates within the host body cavity (Stewart Merrill and Cáceres, 2018), and spores are released into the water after host death (Ebert, 2005) either as the cadaver decays or as a result of predation (Cáceres *et al*., 2009; Duffy, 2009).

Within the communities studied, *D. dentifera* and *C. dubia* are commonly infected hosts. However, these hosts are likely different selective environments for *M. bicuspidata* and potentially impact its diversity at the within-host or lake level. Importantly, the hosts differ in body size at maturity, with *C. dubia* adults being ~1 mm and *D. dentifera* adults being ~1.5–2.5 mm (Dodson *et al*., 2010), which could affect parasite infection or spore production (Auld *et al*., 2017). Indeed, within *D. dentifera*, *M. bicuspidata* produces more spores in larger hosts (Hall *et al*., 2009; Penczykowski *et al*., 2014; Civitello *et al*., 2015), likely due to space and/or resource constraints. Additional traits that affect infection such as spore capture during feeding, penetrability of the gut epithelium, or immune responses could also differ between the host species. In previous lab assays, infectivity and spore production was substantially lower in *C. dubia* than in *D. dentifera* (Strauss *et al*., 2015; Auld *et al*., 2017). However, in those studies, spores were sourced only from infected *D. dentifera* (i.e. collected by grinding up infected *D. dentifera* hosts) rather than *C. dubia* hosts. Although *D. dentifera* and *C. dubia* co-occur in many lakes (Tessier and Woodruff, 2002; Hall *et al*., 2010), their habitat preferences differ (Desmarais and Tessier, 1999; Strauss *et al*., 2016), so abundances of the two hosts vary. Parasite genetic diversity could thus be influenced by the distribution of hosts in lakes across a landscape.

### Field survey

In order to quantify outbreak size in *D. dentifera* and *C. dubia*, we surveyed 15 lakes near Ann Arbor, Michigan and 35 lakes in Greene and Sullivan Counties, Indiana. Lakes were sampled approximately every 2 weeks from mid-July until mid-November 2015 by combining three vertical plankton tows from different locations in the deepest part of the lake. These live samples were subsampled within 36 h of collection until at least 200 *D. dentifera* and all *C. dubia* in those subsamples were counted and diagnosed visually (under a dissecting microscope) for infection with *M. bicuspidata*; hosts were diagnosed as infected if they contained asci, indicating they were fully infected (Stewart Merrill and Cáceres, 2018). To quantify outbreak size, we calculated area under the infection prevalence time series for each host and lake using the trapezoid rule (Penczykowski *et al*., 2014), thus units for this metric are prevalence × days. A linear model was used to test the association between outbreak sizes in the two host species.

### Sample collection and genotyping

We evaluated genetic structure of parasite populations using microsatellites. We genotyped *M. bicuspidata* from 51 infected hosts collected from five lakes in Livingston and Washtenaw counties, Michigan, and 11 lakes in Greene and Sullivan counties, Indiana, in July–November of 2015 ( Tables S1 and S2). To create primers to amplify microsatellite regions, we located simple sequence repeats (with the MISA script; Thiel, 2003) in the *M. bicuspidata* genome (Ahrendt *et al*., 2018) and then used Primer 3 software (Rozen and Skaletsky, 2000). Out of 24 candidate primer pairs, we selected nine that gave the most consistent amplification and variation between samples (Table S3). DNA extraction from infected *D. dentifera* and *C. dubia* and genotyping followed standard methods (see the Supplementary material).

Population genetic metrics were calculated using the R package poppr version 2.8.2 (Kamvar *et al*., 2014; see the Supplementary material). We calculated Prevosti genetic distance between each parasite sample: the fraction of allelic differences between two parasite genotypes out of all loci (Wright, 1978). With these distances we constructed a dendrogram using the unweighted pair group method with arithmetic mean (UPGMA). We generated support for each node using 1000 bootstrapped samples (Kamvar *et al*., 2014). The dendrogram allows for a visual inspection of how the diversity of *M. bicuspidata* genotypes are organized and if organization depends on host species, region (IN or MI), or lake. Then, to determine if host species, region, or lake was statistically associated with the structure of the parasite populations, we ran analyses of molecular variance (AMOVA) with the Prevosti distances among genotypes. In an AMOVA, genotypes are grouped into hierarchical categories (here: host species, region, and lake), and the significance of the similarity of genotypes in each category is tested (Excoffier *et al*., 1992). Since there was not an obvious hierarchy of categories in our study, we performed two AMOVAs. The first (AMOVA 1) designated host species as the highest level of hierarchy followed by region and lake. The second (AMOVA 2) designated region as the highest level of hierarchy followed by lake and host species.

### Cross-infection experiment

Because *M. bicuspidata* infects both *D. dentifera* and *C. dubia* in nature, we tested if the parasite was equally successful infecting each host species with a cross-infection experiment. We quantified infectivity and spore production of parasites collected from *D. dentifera* and *C. dubia* in host clones of each species. For clarity we refer to animals exposed in the experiment as ‘exposed hosts’ and animals from which parasites were isolated for the experiment as ‘source hosts’. From our genotyping results, it seemed likely that cross infection patterns might differ for parasites collected from different lakes. Therefore, cross infection trials for parasites from different lakes were performed and analysed separately.

In September 2017, we used *D. dentifera* and *C. dubia* collected from plankton tows to establish unparasitized asexual isofemale lines from several lakes that we thought might have *M. bicuspidata* outbreaks in both hosts later in the fall. However, only one of these (Benefiel) ended up having an outbreak in both host species. Thus, in November, we also established asexual isofemale lines (hereafter: ‘clones’) from Goose Lake, where an outbreak of *M. bicuspidata* was occurring in both host species. We used plankton tows collected from Benefiel Lake and Goose Lake in November 2017 to collect infected animals to be used as the source of *M. bicuspidata* spores from *D. dentifera* and *C. dubia* hosts for experimental infections. With these, we created spore slurries by homogenizing infected animals. For Benefiel Lake, we created one spore slurry by pooling infected *D. dentifera* and a second spore slurry by pooling infected *C. dubia.* Then, two to four groups of six 7-day old individuals of a given clone (5 *D. dentifera* clones and 5 *C. dubia* clones; Table 1) were exposed to 250 parasite spores/ml from the *D. dentifera*-sourced slurry or the *C. dubia*-sourced slurry. We performed the Goose Lake experiment in a similar fashion but in two blocks, with each block having different spore slurries composed of either infected *D. dentifera* or infected *C. dubia.* Due to difficulties growing up individuals of clones from both lakes, exposures were imbalanced, but this was especially the case for Goose Lake, since we had less time to grow up clones; we exposed zero to six group(s) of a given clone (5 *D. dentifera* clones and 5 *C. dubia* clones; Table 1) to the spore slurries. All exposures lasted 48 hours and took place in 80 mL of filtered (with A/E 1 μm filters, Pall) water from a lake near Ann Arbor, MI (North Lake). We routinely use filtered water from this lake for culturing *Daphnia* spp. and *C. dubia* and have never had animals become infected unintentionally (i.e. in a beaker to which we had not added *Metschnikowia* spores). On the day of exposure, we added algal food, 12 500 cells *Ankistrodesmus falcatus*/mL (‘AJT’ strain; Schomaker and Dudycha, 2021), to each beaker. On the second day of exposure, an additional 18 750 cells *Ankistrodesmus falcatus*/mL were added to each beaker. After exposure and twice weekly thereafter, exposed animals were moved to 100 mL spore-free filtered lake water and fed 25 000 cells *Ankistrodesmus falcatus*/mL daily (at 20°C with a 16:8 h light:dark cycle). Hosts were fed less food during exposure because this increases infection (Hall *et al*., 2007); afterwards, hosts were fed saturating food levels.

**Table 1.** Number of replicate beakers exposed to *Metschnikowia bicuspidata* from each isolation host

Experimental clone name	Host	Number of replicates exposed to *M. bicuspidata* from *Daphnia Dentifera*	Number of replicates exposed to *M. bicuspidata* from *Ceriodaphnia dubia*
BenefielDaphnia4	*D. dentifera*	3	3
BenefielDaphnia6	*D. dentifera*	3	3
BenefielDaphnia7	*D. dentifera*	2	2
BenefielDaphnia14	*D. dentifera*	3	3
BenefielDaphnia16	*D. dentifera*	3	3
BenefielCerio13	*C. dubia*	3	4
BenefielCerio6	*C. dubia*	4	4
BenefielCerio10	*C. dubia*	2	2
BenefielCerio1	*C. dubia*	4	4
BenefielCerio15	*C. dubia*	4	4
GooseCerioB	*C. dubia*	4/6	4/2
GooseCerioA	*C. dubia*	4/3	3/3
GooseCerioC	*C. dubia*	4/2	3/1
GooseCerioI	*C. dubia*	3/6	3/2
GooseCerioJ	*C. dubia*	3/2	3/1
GooseDaphniaA	*D. dentifera*	1/1	1/1
GooseDaphniaH	*D. dentifera*	1/5	1/4
GooseDaphniaE	*D. dentifera*	1/0	1/1
GooseDaphniaD	*D. dentifera*	0/0	0/1
GooseDaphniaC	*D. dentifera*	0/0	0/1

The experiment with Goose Lake hosts and spores was completed in two blocks; the number before the slash indicates the numbers of beakers in the first block and the number after the slash indicates numbers of beakers in the second block.

After 11 days, we diagnosed exposed hosts with a dissecting microscope; as with the field survey, animals were considered infected if they contained asci (Stewart Merrill and Cáceres, 2018). We ended the experiment before natural host death; death rates in natural populations indicate that hosts are likely to die from factors like predation prior to dying from virulent effects of parasites (Duffy and Hall, 2008), and spores remain infectious after infected hosts are killed by predators (Cáceres *et al*., 2009; Duffy, 2009).

Infected individuals from the experiment were frozen for later processing, which involved spore counts, measuring spore length, and genotyping. First, we counted spores: each infected experimental animal was homogenized in 50 *μ*L of water for 30 s with a battery-powered pestle. Three 10 *μ*L aliquots of the homogenized solution were placed on a hemocytometer and spores within the grid were counted under 400× magnification. Average counts were used to quantify spore yields per infected individual. We then measured the length of a random sampling of spores from each infected individual: for each counted grid, one photograph was taken of spores at 400× magnification with a microscope camera (DP73, Olympus). The spores in view were measured with cellSens software (Olympus), and average spore length was computed across all three photographs. On average, 14.9 spores were measured per infected animal although this ranged from 3 to 38 spores. Finally, we genotyped *M. bicuspidata* from a subset of the homogenized infected hosts (42 and 12 from the Benefiel and Goose cross-infection experiments respectively) in order to determine which parasite genotype was responsible for infection with similar methods to the genotyping study (see the Supplementary material).

We analysed experimental results (i.e. proportion infected and number and length of spores) for each lake separately with generalized linear mixed effects models or linear mixed effects models using the lme4 package version 1.1.21 (Bates *et al*., 2015). Proportion infected (binomial errors) and number and length of spores produced (Gaussian errors) were each modelled with an interaction between exposed host and source host (fixed effects) and with host clone included as a random effect. Beaker was included as an additional random effect for the latter two analyses where metrics were from infected individuals, to account for potential non-independence of individuals that were in the same beaker. Non-significant interactions were dropped. The experimental cross infections using spores from Goose Lake were completed in two temporal blocks (adding another random effect to the analysis for the Goose Lake experiment; Table 1). *Post hoc* comparisons were computed using the emmeans package version 1.3.3 (Lenth, 2016). We used a linear mixed effects model to describe the number of spores produced in an infection as a function of the interaction between mean spore length and exposed host species, with clone, beaker, and block (for the Goose experiment) as random effects.

## Results

### Field survey

**Fig. 1.**
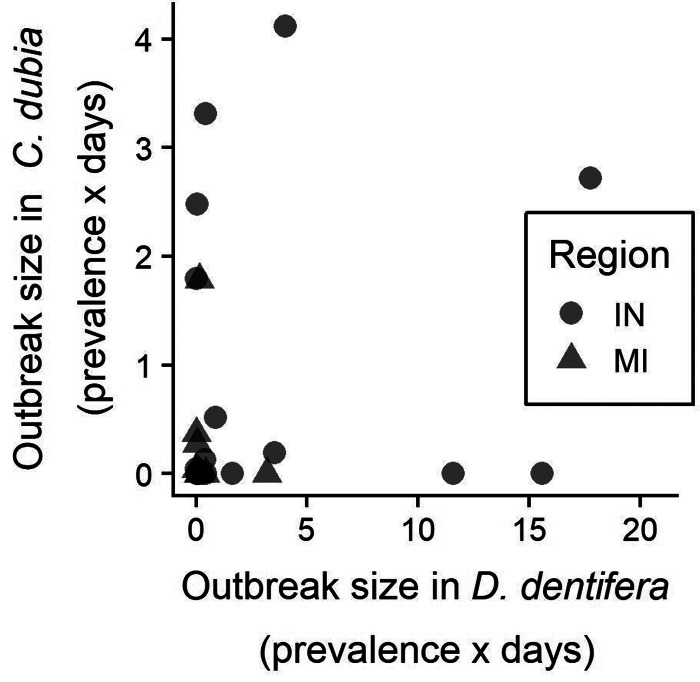
Sizes (time-integrated prevalence) of *M. bicuspidata* outbreaks in 2015 in *D. dentifera* and *C. dubia* were not correlated. Points are partially transparent to allow better visualization of overlapping points. Data are only plotted for lake-years where both hosts were present at some point during the sampling period.

Outbreaks of *M. bicuspidata* tended to occur in either *D. dentifera* or *C. dubia*, but not in both in the same year (Fig. 1). Outbreak size in one host species was not correlated with outbreak size in the other host (*F*_1,25_ = 0.904, *P* = 0.351).

### *Metschnikowia bicuspidata* genotypes

**Fig. 2.**
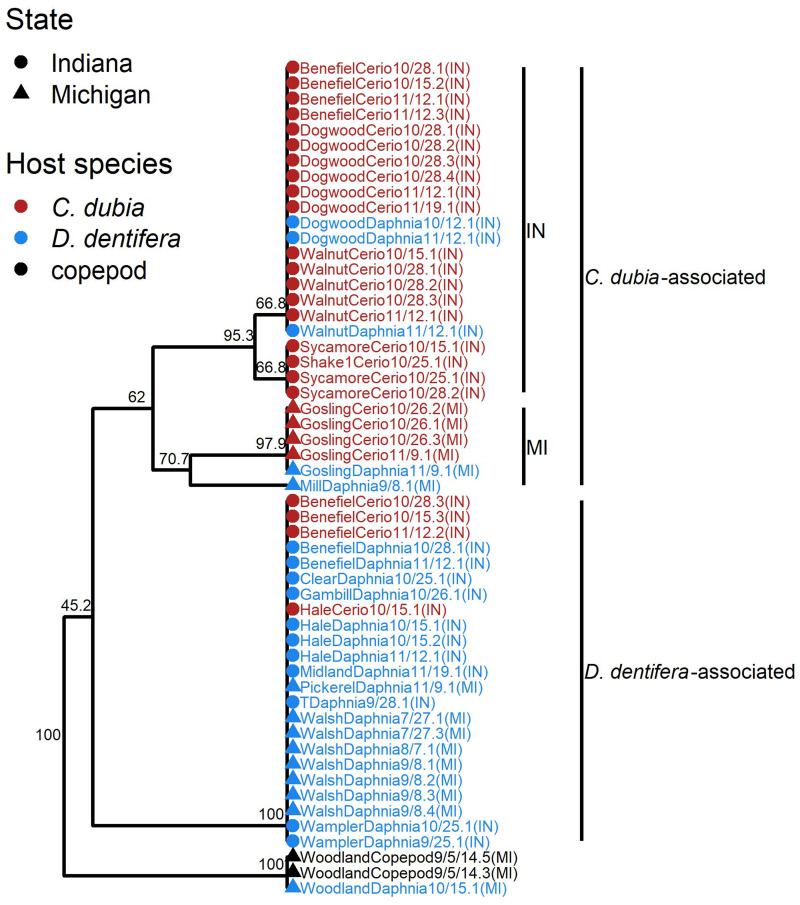
Microsatellite genotyping of *M. bicuspidata* from infected *D. dentifera* (blue font) and *C. dubia* (red font) collected in fall 2015 in Indiana (IN) and Michigan (MI) lakes (USA). Genotypes of *M. bicuspidata* infecting two copepods collected in fall 2014 are also included. We found three parasite clades. Of these, two were particularly common, with one primarily infecting *D. dentifera* and the other primarily infecting *C. dubia*. Within the *C. dubia*-associated clade, genotypes fall into different clades in IN and in MI. Tip labels follow the format LakeHostDate.Replicate(State). See supplemental Table S1 for a list of samples. Scale bar indicates Prevosti distance between individuals. Bootstrap support (>40%) is noted on nodes. Source hosts used in the lab experiments were collected in a subsequent year and pooled in spore slurries (see Materials and methods); thus, individual source hosts were not genotyped and are not on the dendrogram.

We found six parasite genotypes infecting *D. dentifera* and *C. dubia* hosts in our survey lakes, grouped within three distinct clades (Fig. 2). We found an average of 2.78 alleles per locus, and the six genotypes differed on average at 5.3 loci. Nei's gene diversity (*H*_S_) measures the probability that two randomly drawn alleles from a given locus in a population will be different. Over all parasites isolated from *D. dentifera* and *C. dubia*, H_S_ was 0.409 [95% confidence interval (CI): (0.379, 0.422)], but for parasites infecting each host species, H_S_ was lower [*D. dentifera H*_S_ = 0.291, 95% CI: (0.222, 0.333); *C. dubia H*_S_ = 0.290, 95% CI: (0.232, 0.324)], indicating lower diversity of genotypes infecting each individual host species.

We calculated the index of association, *I*_A_, among alleles in clone corrected (data were filtered so that each multilocus genotype was represented once) parasite genotypes to evaluate if parasites were outcrossing or clonal (Smith *et al.*, 1993). The clone corrected index of association was 0.995 (*P* = 0.007) indicating that *M. bicuspidata* reproduces clonally.

Of the three most abundant *M. bicuspidata* genotypes, one genotype was present in both regions and found primarily infecting *D. dentifera* (Fig. 2; the single genotype in the *D. dentifera-*associated clade). The other two abundant *M. bicuspidata* genotypes were found primarily in *C. dubia* with one genotype common in Indiana lakes and the other genotype common in Michigan lakes (Fig. 2; the two most common genotypes in the *C. dubia*-associated clade). However, none of the three most prevalent *M. bicuspidata* genotypes was restricted to a single host species.

There were also three less common *M. bicuspidata* genotypes. One was found in Sycamore Lake and Shake 1 Lake (both in Indiana). Sycamore Lake only had infections in *C. dubia,* and Shake 1 Lake had low infection levels in *D. dentifera* early in the season, but not when samples were collected. The other two less common *M. bicuspidata* genotypes were found infecting hosts in Michigan lakes, Woodland and Mill. In both of these lakes, it is possible that these infections spilled over from other host species. In Woodland Lake, two copepods collected in 2014 were infected by the same *M. bicuspidata* genotype as an infected *D. dentifera* that was collected in 2015 (Fig. 2). Marine copepods have previously been found to be infected with a different species of *Metschnikowia* (Seki and Fulton, 1969); this is the first published record of *M. bicuspidata* in copepods, although we have seen *M. bicuspidata* in copepods during other sampling of Indiana Lakes, as well (S.R. Hall, personal observation). In Mill Lake, only two infected *D. dentifera* were counted over the entire season. Although outbreaks didn't take off in any species, one infected *C. dubia*, one infected *D. ambigua*, and two infected *D. retrocurva* were also documented in this lake during fall 2015, but parasites infecting these animals were not genotyped.

Overall, parasite genotypes from infected *D. dentifera* and *C. dubia* clustered by host species, although occasionally individuals of different host species in the same lake shared the same parasite genotype, showing that each parasite genotype can infect both hosts (Fig. 2). When host species was the highest level of hierarchy (AMOVA 1), host species groups explained 32.17% of the variation between samples (*P* = 0.001, Table 2), but when it was the lowest level (AMOVA 2) it only explained 6.29% of the variation between samples (*P* = 0.177, Table 2) with lake groups accounting for 73.37% of the variation (*P* = 0.011, Table 2). Together, the AMOVAs suggest genetic structure: *D. dentifera* and *C. dubia* tended to get infected by different *M. bicuspidata* genotypes when collected from different lakes. Within lakes, there was often transmission of a given *M. bicuspidata* genotype between the host species.

**Table 2.** Hierarchical analysis of variance suggests genotypic variance is partitioned by host and lake

AMOVA 1				
	Observed partition			
Variance component	Variance	% total	ϕ-statistics	*P*^a^
Between hosts	1.51	32.17	ɸ_Host−Total_ = 0.32	(greater) 0.001
Between states	0.53	11.26	ɸ_State−Host_ = 0.17	(greater) 0.167
Between lakes	2.00	42.47	ɸ_Lake−State_ = 0.75	(greater) 0.001
Within lakes	0.66	14.10	ɸ_Lake−Total_ = 0.86	(less) 0.001
AMOVA 2				
	Observed partition			
Variance component	Variance	% total	ɸ-statistics	*P*^a^
Between states	0.16	3.98	ɸ_State−Total_ = 0.04	(greater) 0.301
Between lakes	2.98	73.37	ɸ_Lake−State_ = 0.76	(greater) 0.011
Between hosts	0.26	6.29	ɸ_Host−Lake_ = 0.28	(greater) 0.177
Within hosts	0.66	16.36	ɸ_Host−Total_ = 0.84	(less) 0.001

The two AMOVA analyses were designed to test if genetic variance was organized by host species, region (IN or MI), or lake using a hierarchical approach. AMOVA 1 designates host species as highest level of the hierarchical analysis followed by region and lake. AMOVA 2 designates region as the highest level followed by lake and host species.

a The *P* values are calculated by 999 random permutations of the distance matrix (composed of Prevosti distances) between genotyped parasites. Significance is attained if the observed ɸ-statistic (and variance component) is greater or less (noted in parentheses) than it would be by chance (Excoffier *et al*., 1992).

### Cross-infection experiment

**Fig. 3.**
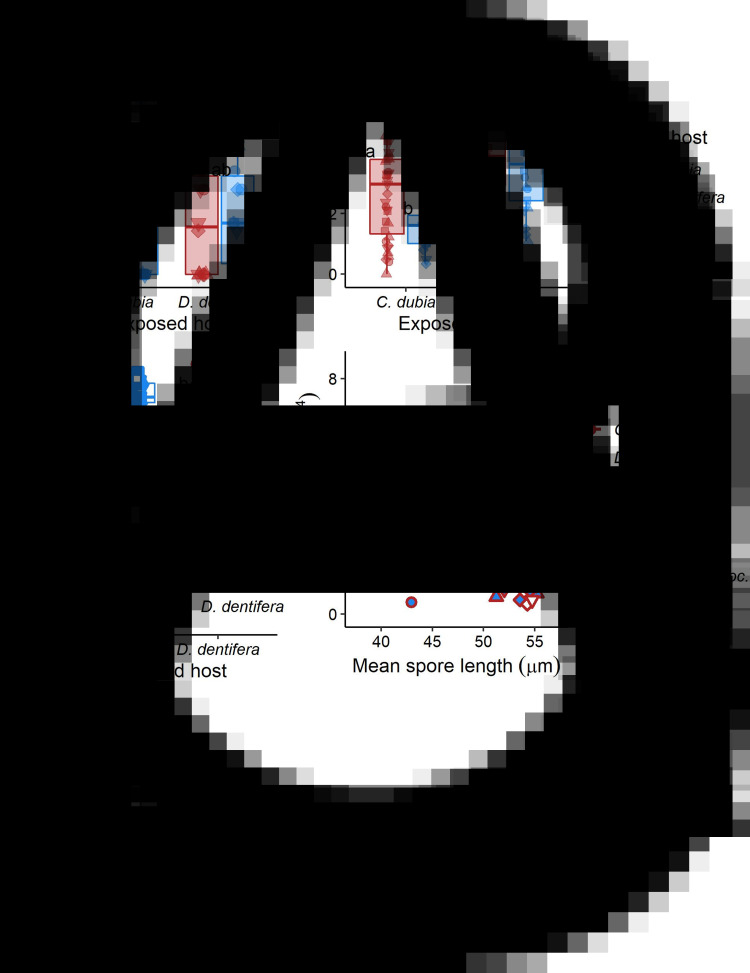
The combination of exposed and source hosts from Benefiel (IN) mattered for infection and spore production. (A) The proportion of infected animals depended on an exposed × source host interaction: *C. dubia* were most infected by *C. dubia*-sourced spores. Points represent beakers, and shapes represent different experimental host clones. (B) More spores were produced in exposed *D. dentifera* hosts; in exposed *C. dubia*, more spores were produced when infected with *C. dubia*-sourced *M. bicuspidata*. (C) Spores in *C. dubia* exposed hosts were smaller when sourced from *C. dubia*. The smaller spores belonged to the *C. dubia*-associated *M. bicuspidata* genotype (red fill) found in Benefiel in 2015, while the larger spores belonged to the *D. dentifera*-associated *M. bicuspidata* genotype (blue fill). (D) When infected with the *C. dubia*-associated genotype, exposed *C. dubia* hosts produced a relatively large number of small spores (red border-red fill symbols); in contrast, when infected with the *D. dentifera*-associated genotype, exposed *C. dubia* hosts produced fewer and larger spores (red border-blue fill symbols). Exposed *D. dentifera* hosts (blue border) only produced relatively large spores. Within exposed *D. dentifera*, animals that had larger spores also produced more spores. In (B)–(D) points represent individual infected hosts with shapes designating different experimental host clones. Beaker was also included as a random effect in statistical models.

The results of our cross-infection experiment differed between the two lakes. In the cross-infection experiment using exposed and source hosts from Benefiel Lake, infection and spore production depended on both the source host species and the exposed host species. The proportion of infection in *C. dubia* was higher when exposed to *C. dubia*-sourced spores, as compared to *D. dentifera*-sourced spores (Fig. 3A; source × exposed host interaction: LRT = 8.82, *P* = 0.003; post-hoc comparison of prevalence in *C. dubia* for *C. dubia*- *vs D. dentifera*-sourced spores: *z* = 3.18, *P* = 0.008). In contrast, prevalence of infection in *D. dentifera* was consistent when they were exposed to *C. dubia-*sourced spores and *D. dentifera*-sourced spores (Fig. 3A).

Spore production at 11 days post infection also depended on source and exposed hosts (source host: LRT = 8.86, *P* = 0.003; exposed host: LRT = 6.77, *P* = 0.009; Fig. 3B). In exposed *C. dubia, C. dubia*-sourced *M. bicuspidata* produced more spores than *D. dentifera*-sourced *M. bicuspidata* (post-hoc: *t*-ratio = 2.81, *P* = 0.04; Fig. 3B). In exposed *D. dentifera*, spore production at 11 days did not differ significantly between animals infected by *C. dubia-*sourced and *D. dentifera*-sourced spores (Fig. 3B).

The size of spores produced in infections depended on source and exposed host (Fig. 3C; LRT = 25.46, *P* < 0.001): *C. dubia*-sourced *M. bicuspidata* produced smaller spores in exposed *C. dubia* hosts as compared to spores produced in exposed *D. dentifera* hosts sourced from either host species (post-hoc: from *D. dentifera*: *t*-ratio = −8.94, *P* < 0.001; from *C. dubia: t*-ratio = −7.77, *P* < 0.001) and to spores produced in *C. dubia* when sourced from *D. dentifera* (post-hoc: *t*-ratio = −8.69, *P* < 0.001). These smaller spores belonged to the most prevalent Indiana (IN) *C. dubia-*associated genotype (i.e. in the *C. dubia*-associated clade) in the 2015 survey (Fig. 2). In contrast, the larger spores belonged to the main *D. dentifera-*associated genotype. *Ceriodaphnia dubia* exposed to *M. bicuspidata* sourced from *C. dubia* became infected by both genotypes, whereas the *D. dentifera* exposed to spores sourced from *C. dubia* only became infected by the main *D. dentifera-*associated genotype (Fig. 3C). Furthermore, *C. dubia* produced more spores when infected by the smaller-spored genotype, as compared to when they were infected by the larger-spored genotype (Fig. 3D; spore size × host species: LRT = 15.97, *P* < 0.001). In summary, *C. dubia* source hosts from Benefiel must have been infected by both genotypes when they were collected from the field. Then, in the experiment, exposed *C. dubia* hosts became infected by both genotypes; in contrast, *D. dentifera* only became infected by the larger, *D. dentifera*-associated genotype.

**Fig. 4.**
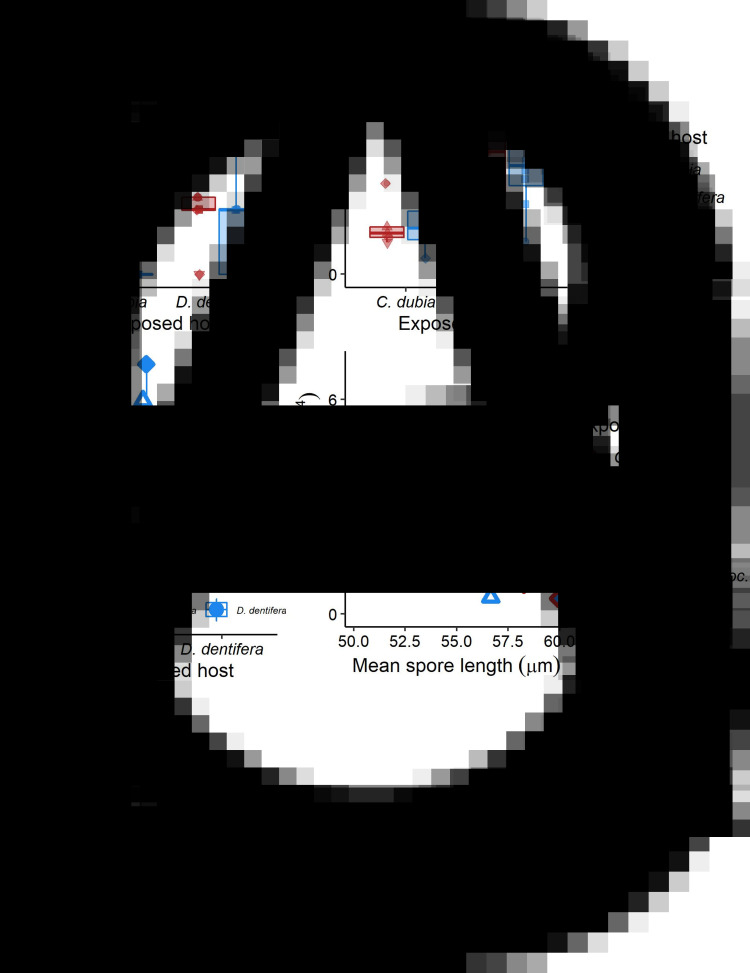
The combination of exposed and source hosts yielded different results shown here from Goose lake than from Benefiel Lake, likely because this lake harboured only the *D. dentifera*-associated *M. bicuspidata* genotype. (A) No influence of source or exposed host on infection rates could be detected. Points represent beakers, and shapes represent different experimental host clones. (B) More spores were produced in *D. dentifera* hosts, but the origin of spores did not affect spore production. (C) There was no significant difference in spore size between the exposed groups. Furthermore, all genotyped infections belonged to the *D. dentifera-*associated genotype. (D) Spore length did not significantly influence spore production in either *D. dentifera* or *C. dubia,* likely because all spores were large relative to spores of the *C. dubia*-associated genotypes. In (B)–(D) points represent individual infected hosts with shapes designating different experimental host clones. Beaker was also included as a random effect in statistical models.

Results from the cross infection with hosts and parasites from Goose Lake showed different patterns. Overall infection levels were low, and therefore no influence of source or exposed host on infection rates could be detected (Fig. 4A). More spores were produced in *D. dentifera* hosts (LRT = 3.84, *P* = 0.05; Fig. 4B), although there was no difference in spore quantities produced by *M. bicuspidata* from the two source host species (LRT = 0.00, *P* = 0.98). Spore sizes were not significantly different between the groups (exposed species: LRT = 0.55, *P* = 0.46; source species: LRT = 0.59, *P* = 0.44; Fig. 4C). Notably, all genotyped samples belonged to the *D. dentifera-*associated genotype (Fig. 4C and D). Infection by only one *M. bicuspidata* genotype is consistent with the lack of a source host effect on infection rate, spore yield, and spore size on exposed host species in this lake.

## Discussion

*Metschnikowia bicuspidata* is a widespread parasite of *Daphnia* (Green, 1974; Ebert, 2005) with substantial impacts on the ecology (Duffy, 2007; Duffy and Hall, 2008; Penczykowski *et al*., 2020) and evolution (Duffy and Sivars-Becker, 2007; Duffy *et al*., 2008, 2012) of its hosts. As reviewed in the introduction, prior studies failed to detect phenotypic or genetic variation in *Daphnia* hosts, even though outbreaks are large, common, and occur in multiple hosts on multiple continents. Here, using more sensitive techniques, we found significant intraspecific variation in *M. bicuspidata*. We found six parasite genotypes grouped within three distinct clades. One of these clades was rare (but included a *M. bicuspidata* genotype that infected copepods and *D. dentifera* – notable given that the most recent common ancestor of these taxa lived ~550 MYA during the Cambrian Era (Schwentner *et al*., 2017)). Of the two main parasite clades, one was primarily associated with *D. dentifera* and the other was primarily associated with *C. dubia*. In lake populations, outbreaks tended to occur in one species or the other. However, each of these genotypes could be found in both hosts within a lake, indicating that parasite genotypes were not completely restricted to the host species with which they were most commonly associated. In laboratory cross-infection experiments, infection outcomes depended on the lake from which parasite spores were collected, likely because only one of the two lakes contained the *C. dubia*-associated genotype. In the experiment where spores were collected from this lake (Benefiel), the *D. dentifera-*associated genotype was able to infect both host species, but produced fewer spores at 11 days post infection in *C. dubia* hosts than the *C. dubia-*associated genotype did. In contrast, the *C. dubia-*associated genotype did not infect *D. dentifera*. The *C. dubia*-associated genotype produced smaller spores, as compared to the *D. dentifera* genotype, even when they both infected the same host species, *C. dubia*. In the experiment where spores were collected from the other lake (Goose), there was a lack of effect of source host on infection rate, spore yield, and spore size, which is consistent with only the *D. dentifera*-associated genotype causing infection in this lake.

**Fig. 5.**
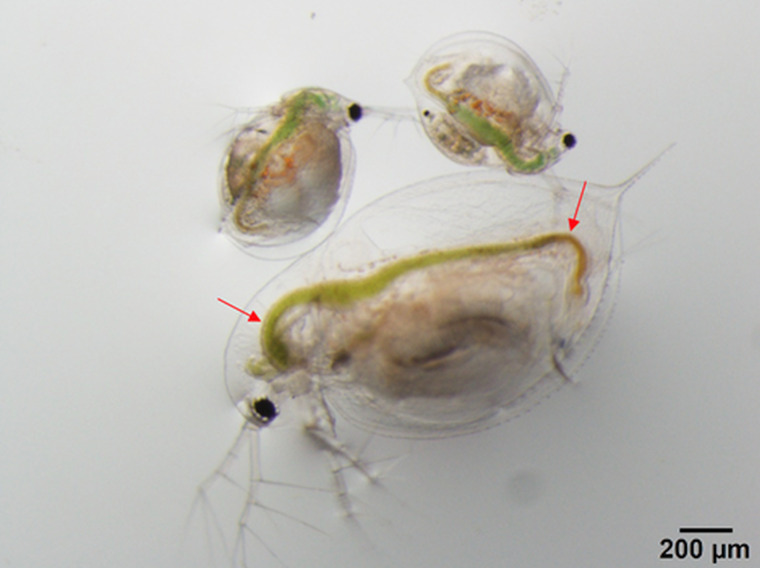
Although both *D. dentfera* and *C. dubia* grow continuously, adult *D. dentifera* are larger than adult *C. dubia*, potentially influencing competence for the parasite. The photograph shows two adult female *C. dubia* (on top) and one adult female *D. dentifera* (below). Arrows show bends where spores most likely pierce the gut wall (Stewart Merrill and Cáceres, 2018). Photo credit: Meghan A. Duffy.

We hypothesize that spore size might influence the ability of *M. bicuspidata* to infect different hosts, by influencing the likelihood of encountering a spore and/or the probability of infection given encounter. First, the likelihood of encountering a spore will vary based on both filtering rate (Burns, 1969) and feeding appendage structure (Geller and Müller, 1981), both of which correlate with body size. Second, once a spore is encountered, infection is a mechanical process in which spores penetrate the host's gut wall (Stewart Merrill and Cáceres, 2018; Stewart Merrill *et al*., 2020); one possibility is that size could impact the probability of piercing through this barrier. Infection usually begins at the anterior or posterior bends in the gut where long, needle-like spores may ram straight into the gut wall instead of making the ‘turn’ with the rest of the gut contents (Stewart Merrill and Cáceres, 2018; Fig. 5). Smaller spores may lodge in the gut for smaller animals; however, in larger animals, small spores would more easily flow around the bend in the gut without piercing the gut wall (since gut volume scales with host body volume; Hall *et al*., 2007). Although both *C. dubia* and *D. dentifera* grow continuously and show variation in adult body size, *C. dubia* is smaller-bodied than *D. dentifera* (Dodson *et al*., 2010; Fig. 5). If either or both of these mechanisms (likelihood of encounter and infection given encounter) is operating, it would suggest that differences in host species composition and/or stage structure could influence the fitness of different *M. bicuspidata* genotypes.

Interestingly, this is not the first study to find variation in spore size in *Metschnikowia*. An earlier study also found two sizes of *M. bicuspidata* spores infecting *Daphnia magna* and *Daphnia pulex* in southern England (Stirnadel and Ebert, 1997). In addition, a different *Metschnikowia* species, *Metschnikowia typographi*, that infects bark beetles also has different size morphs that may be specialized on different bark beetle species (Weiser *et al*., 2003; Yaman and Radek, 2008). More extensive sampling (both geographically and in terms of host species) and genotyping could help us to understand the evolutionary relationships between the genotypes documented in this study as well as the evolutionary history of *M. bicuspidata* spore size. At present, it is intriguing that, even though we found a relatively small number of genotypes, genotype seems to be associated with *M. bicuspidata* spore size, suggesting adaptation to divergent selection imposed by different hosts. This adaptation could be facilitated by the apparent reproductive isolation (through clonality or strict selfing) between *M. bicuspidata* genotypes despite the great potential for interbreeding (i.e. co-occurrence of the major clades in the same lake and the long-distance dispersal detected here). Spore size is associated with both neutral markers and virulence in other fungal parasites (Fisher *et al*., 2009). Fisher *et al*. (2009) also noted the surprising link of *Bd* genotype with functional traits, where, as is also true for *M. bicuspidata*, molecular markers show low genetic diversity. The observation of traits diverging faster than neutral markers suggests they are under strong selection.

Parasite fitness will depend not only on the likelihood of infecting a host, but also on the spore yield from that host. In our cross-infection experiments, the larger-spored *D. dentifera*-associated genotype of *M. bicuspidata* was able to infect both species, but it produced fewer spores in *C. dubia* (on average less than half as many spores in *C. dubia* hosts than in *D. dentifera* hosts at 11 days post infection). One possibility is that fewer of these large spores can be produced in smaller *C. dubia* due to limitations on space and/or resources. In general, parasite biomass has been shown to scale with host body mass (Poulin and George-Nascimento, 2007). Several other parasites produce fewer transmission stages in smaller hosts including the bacterium *Pasteuria ramosa* in *Daphnia* (Cressler *et al*., 2014; Clerc *et al*., 2015) and the microsporidian *Nosema whitei* in *Tribolium* beetles (Blaser and Schmid-Hempel, 2005). Overall, spore yield of *C. dubia-*associated *M. bicuspidata* is higher in *C. dubia*, and spores from *C. dubia* are more likely to infect *C. dubia* (as compared to spores from *D. dentifera*).

Given these differences in infectivity and spore yield, which parasite genotypes are favoured will depend on the relative densities of the two host species. The *D. dentifera*-associated genotype was able to infect both host species in our laboratory cross-infection experiment but had lower spore yield in *C. dubia*. The *C. dubia*-associated genotype did not infect *D. dentifera* in our lab experiment (but was found in *D. dentifera* in the field); however, *C. dubia* infected with the *C. dubia-*associated genotype produced many more spores as compared to *C. dubia* infected with the *D. dentifera*-associated genotype. Thus, which genotype of *M. bicuspidata* is favoured will likely depend on which host species dominates in a given lake, which is driven by a suite of biotic and abiotic factors (Desmarais and Tessier, 1999; Tessier and Woodruff, 2002). Further study of the impacts of these factors on parasite distributions could yield insights into why Goose Lake apparently did not host the *C. dubia*-associated genotype despite having a large enough outbreak in *C. dubia* hosts that we were able to collect enough infected *C. dubia* for our experiment (a task that proved difficult in many lakes, see ‘Materials and methods’). Future studies on the evolution of *M. bicuspidata* would also be interesting, as evolution will likely depend on both trade-offs faced by genotypes as well as on the relative quantity and quality of the different hosts (Gandon, 2004).

By studying two zooplanktonic host species in two regions, we have uncovered diversity in an ecologically important parasite that was previously thought to harbour little or no genetic diversity. Parasite genotypes clustered by host species and by lake and differed in spore size and cross-species transmission. Future studies should further explore the causes and consequences of the association between parasite spore size, host body size, and the likelihood of interspecific transmission, as this may help explain patterns of infection in this ecologically important system.

## Supplementary material

For supplementary material accompanying this paper visit https://doi.org/10.1017/S0031182021000949. click here to view supplementary material
