# Supplementary material for: Genotypic variation in an ecologically important parasite is associated with host species, lake and spore size
Source: Parasitology. 2021 Jun 9;148(11):1303–12. doi: 10.1017/S0031182021000949 (PMC8383271; doi:10.1017/S0031182021000949)
Supplement: Supplementary file 1 [file S0031182021000949sup001.docx]

**Genotypic variation in an ecologically important parasite is associated with host species, lake, and spore size**

**SUPPLEMENTAL INFORMATION**

**Table S1.** List of genotyped parasites for Figure 1 in the main text.

| **Sample** | **Host** | **Lake** | **Date** |
| --- | --- | --- | --- |
| BenefielCerio10/15.2(IN) | *C. dubia* | Benefiel (IN) | 10/15/15 |
| BenefielCerio10/15.3(IN) | *C. dubia* | Benefiel (IN) | 10/15/15 |
| BenefielCerio10/28.1(IN) | *C. dubia* | Benefiel (IN) | 10/28/15 |
| BenefielCerio10/28.3(IN) | *C. dubia* | Benefiel (IN) | 10/28/15 |
| BenefielCerio11/12.1(IN) | *C. dubia* | Benefiel (IN) | 11/12/15 |
| BenefielCerio11/12.2(IN) | *C. dubia* | Benefiel (IN) | 11/12/15 |
| BenefielCerio11/12.3(IN) | *C. dubia* | Benefiel (IN) | 11/12/15 |
| BenefielDaphnia10/28.1(IN) | *D. dentifera* | Benefiel (IN) | 10/28/15 |
| BenefielDaphnia11/12.1(IN) | *D. dentifera* | Benefiel (IN) | 11/12/15 |
| ClearDaphnia10/15.1(IN) | *D. dentifera* | Clear (IN) | 10/15/15 |
| DogwoodCerio10/28.1(IN) | *C. dubia* | Dogwood (IN) | 10/28/15 |
| DogwoodCerio10/28.2(IN) | *C. dubia* | Dogwood (IN) | 10/28/15 |
| DogwoodCerio10/28.3(IN) | *C. dubia* | Dogwood (IN) | 10/28/15 |
| DogwoodCerio10/28.4(IN) | *C. dubia* | Dogwood (IN) | 10/28/15 |
| DogwoodCerio11/12.1(IN) | *C. dubia* | Dogwood (IN) | 11/12/15 |
| DogwoodCerio11/19.1(IN) | *C. dubia* | Dogwood (IN) | 11/19/15 |
| DogwoodDaphnia10/12.1(IN) | *D. dentifera* | Dogwood (IN) | 10/12/15 |
| DogwoodDaphnia11/12.1(IN) | *D. dentifera* | Dogwood (IN) | 11/12/15 |
| GambillDaphnia10/26.1(IN) | *D. dentifera* | Gambill (IN) | 10/26/15 |
| GoslingCerio10.26.1(MI) | *C. dubia* | Gosling (MI) | 10/26/15 |
| GoslingCerio10.26.2(MI) | *C. dubia* | Gosling (MI) | 10/26/15 |
| GoslingCerio10.26.3(MI) | *C. dubia* | Gosling (MI) | 10/26/15 |
| GoslingCerio10.26.4(MI) | *C. dubia* | Gosling (MI) | 10/26/15 |
| GoslingDaphniaMale11.9.1(MI) | *D. dentifera* | Gosling (MI) | 11/9/15 |
| HaleDaphnia10/15.1(IN) | *D. dentifera* | Hale (IN) | 10/15/15 |
| HaleDaphnia10/15.2(IN) | *D. dentifera* | Hale (IN) | 10/15/15 |
| HaleCerio10/15.1(IN) | *C. dubia* | Hale (IN) | 10/15/15 |
| HaleDaphnia11/12.1(IN) | *D. dentifera* | Hale (IN) | 11/12/15 |
| MidlandDaphnia11/19.1(IN) | *D. dentifera* | Midland (IN) | 11/19/15 |
| MillDaphnia9/8.1(MI) | *D. dentifera* | Mill (MI) | 9/8/15 |
| PickerelDaphnia10/4.1(MI) | *D. dentifera* | Pickerel (MI) | 10/4/15 |
| Shake1Cerio10.25.1(IN) | *C. dubia* | Shake 1 (IN) | 10/25/15 |
| SycamoreCerio10.15.1(IN) | *C. dubia* | Sycamore (IN) | 10/15/15 |
| SycamoreCerio10.25.1(IN) | *C. dubia* | Sycamore (IN) | 10/25/15 |
| TDaphnia9/28.1(IN) | *D. dentifera* | T (IN) | 9/28/15 |
| WalnutCerio10/15.1(IN) | *C. dubia* | Walnut (IN) | 10/15/15 |
| WalnutCerio10/28.1(IN) | *C. dubia* | Walnut (IN) | 10/28/15 |
| WalnutCerio10/28.2(IN) | *C. dubia* | Walnut (IN) | 10/28/15 |
| WalnutCerio10/28.3(IN) | *C. dubia* | Walnut (IN) | 10/28/15 |
| WalnutCerio11/12.1(IN) | *C. dubia* | Walnut (IN) | 11/12/15 |
| WalnutDaphnia11/12.1(IN) | *D. dentifera* | Walnut (IN) | 11/12/15 |
| WalshDaphnia9/8.1(MI) | *D. dentifera* | Walsh (MI) | 9/8/15 |
| WalshDaphnia9/8.2(MI) | *D. dentifera* | Walsh (MI) | 9/8/15 |
| WalshDaphnia9/8.3(MI) | *D. dentifera* | Walsh (MI) | 9/8/15 |
| WalshDaphnia9/8.4(MI) | *D. dentifera* | Walsh (MI) | 9/8/15 |
| WalshDaphnia7/27.1(MI) | *D. dentifera* | Walsh (MI) | 7/27/15 |
| WalshDaphnia7/27.3(MI) | *D. dentifera* | Walsh (MI) | 7/27/15 |
| WalshDaphnia8/7.1(MI) | *D. dentifera* | Walsh (MI) | 8/7/15 |
| WamplerDaphnia10/25.1(IN) | *D. dentifera* | Wampler (IN) | 10/25/15 |
| WamplerDaphnia9/25.1(IN) | *D. dentifera* | Wampler (IN) | 9/25/15 |
| WoodlandDaphnia10/15.1(MI) | *D. dentifera* | Woodland(MI) | 10/15/15 |
| WoodlandCopepod9/5/14.5(MI) | *Copepod* | Woodland(MI) | 9/5/14 |
| WoodlandCopepod9/5/14.3(MI) | *Copepod* | Woodland(MI) | 9/5/14 |

**Table S2.** Coordinates of study lakes.

| **Lake** | **Coordinates** |
| --- | --- |
| Benefiel | 38.97290585687601, -87.25723674080278 |
| Clear | 39.04946874729811, -87.23895480428229 |
| Dogwood | 38.97564167281498, -87.2580950476674 |
| Gambill | 39.05042692526142, -87.25500514305979 |
| Goose | 39.06171385493238, -87.32381366964393 |
| Gosling | 42.43955173376968, -84.00363301210898 |
| Hale | 38.96970281599828, -87.24556376739183 |
| Midland | 39.12514940346309, -87.17921016502895 |
| Mill | 42.32909371332723, -84.08857253052054 |
| Pickerel | 42.41007682875238, -83.98192985948491 |
| Shake1 | 38.96536513408586, -87.23311831785513 |
| Sycamore | 38.96930242571873, -87.23097255069362 |
| T | 39.02440152786606, -87.25388934422654 |
| Walnut | 38.98164675168223, -87.24290301611154 |
| Walsh | 42.33761525348255, -84.07989002560574 |
| Wampler | 39.017199724854606, -87.22256114310998 |
| Woodland | 42.55897102295908, -83.78895978443465 |

**Table S3.** Primer sequences used for amplifying the 9 loci in *M. bicuspidata* used in this study. Note that all forward primers contain the M13(-21) tail (Schuelke 2000).

| Name | Forward (5’-3’) | | Reverse (5’-3’) |
| --- | --- | --- | --- |
| L3 | TGTAAAACGACGGCCAGTCAAGAGAGACAAGCGGAAGG | | GAACAACACGCTCGCTACAA |
| L7 | TGTAAAACGACGGCCAGTAGGATGCAGGTTTTCTGACG | | TCAGGTGGACTACATTGGCA |
| L8 | TGTAAAACGACGGCCAGTTCGACACACTTCAACGAAGC | | ACAGGTCCTTCAACCTGGTG |
| L9 | TGTAAAACGACGGCCAGTCGTTTCACTAAAAACCCCCA | | TCAGTTGTTTGCCACTGGAC |
| L10 | TGTAAAACGACGGCCAGTGGGTCGTTGATAAGCGAAGA | | TTTAGGGTATTCACGCCGTC |
| L11 | TGTAAAACGACGGCCAGTTGGGTAAATTGTGTGGCAGA | | TTCGACAAAAACGGATCCTC |
| L12 | TGTAAAACGACGGCCAGTCTGCCAGTACTCCTGCATCA | | TTCATCACGTTCGACACCAT |
| L17 | TGTAAAACGACGGCCAGTTACTCGCTCAATGCATCAGG | | CTCCGCAAGGACTTTGCTAC |
| L19 | TGTAAAACGACGGCCAGTATTTGCTCGAGACGCTGTTT | | AATGAAATTGCGGACACACC |
|  | |  |  |

***Supplemental information about DNA extraction, microsatellite amplification, and fragment analysis of samples from the field***

Infected animals collected during natural outbreaks in lakes in Michigan and Indiana were placed individually in microcentrifuge tubes in 90% ethanol. These samples were stored at -20°C until DNA extraction. DNA was extracted with the Mericon DNA Bacterial Plus kit (QIAGEN, Hilden, Germany). Preserved infected animals were placed individually in 200 µL of the provided fast lysis buffer, and a battery powered pestle was used to homogenize each one. Each homogenized sample was transferred to a bead basher tube and vortexed at high speed for 10 minutes. Tubes were then centrifuged and the supernatant of each was saved as the DNA sample. All DNA samples were kept frozen at -20°C until PCR. Genotyping followed standard methods: PCR was performed in 96 well plates with one reaction in each well. A M13(-21) tail was added to each forward primer, and a universal labeled 6FAM M13(-21) primer was used for detection (Schuelke, 2000). PCR reactions were carried out in a final volume of 10 µL with 1X Qiagen multiplex mastermix (QIAGEN, Hilden, Germany), 15 nM forward primer with M13(-21) tails, 500 nM reverse primer, 150 nM labeled 6FAM universal M13(-21) primer, and with 1 µL of DNA. Amplification conditions were: 95°C (15 min), then 35 cycles of 94°C (30 s) / 58°C (3 min) / 72°C (1:30 min), and a final extension at 72°C for 10 min. 1 µl of diluted (1:200) PCR products were added into capillary electrophoresis loading plates containing 11 µl Hi-Di formamide and a LIZ500 size standard. Fragment analysis was performed by the University of Michigan DNA sequencing core, and fragment lengths were read using GeneMapper (ThermoFisher Scientific).

***Supplemental information about genotyping animals from the cross infection experiment***

DNA extraction was performed on the solution remaining from spore counts with the mericon DNA extraction kit (QIAGEN, Hilden, Germany). 200 µl of fast lysis buffer was added to the remaining spore slurry and vortexed. This solution was then transferred to the bead basher tubes, vortexed, centrifuged, and the supernatant was saved as above. We genotyped experimental samples at 8 loci (Supplemental Table S2, excluding L3 because it was monomorphic in all but the Woodland (MI) field samples). Due to lower DNA concentrations in experimental extractions, we altered the PCR recipe to 1X Qiagen multiplex mastermix (QIAGEN, Hilden, Germany), 10 nM forward primer with M13(-21) tails, 400 nM reverse primer, 400 nM 6FAM or HEX labeled universal M13(-21) primer, and 2 µl DNA in 10 µL reactions. Amplification conditions were: 94°C (3 min), then 10 cycles of 94°C (30 s) / 62C-53C (1°C drop each cycle; 30 s) / 72°C (45 sec), followed by 20 cycles of 94°C (30 s) / 53°C (30 s) / 72°C (45 s), followed by 8 cycles of 94°C (30 s) / 53°C (30 s) / 72°C (30 s), and a final extension at 72°C for 30 min. Amplified DNA was diluted 1:100 and loaded into prepared capillary electrophoresis plates with two (one HEX and one 6 FAM labeled) samples per well. DNA levels were much lower in these samples, so not all loci amplified consistently in every sample, but samples were assigned to genotypes discovered in the genotyped natural samples if at least 4 loci were amplified.
